# Performance-Related Material Properties of Asphalt Mixture Components (Second Edition)

**DOI:** 10.3390/ma18214926

**Published:** 2025-10-28

**Authors:** Yao Zhang, Haibo Ding, Yu Chen, Meng Ling

**Affiliations:** 1 College of Civil Engineering and Transportation, Yangzhou University, Yangzhou 225127, China; 2 School of Civil Engineering, Jiulidi Campus, Southwest Jiaotong University, Chengdu 611756, China; haibo.ding@swjtu.edu.cn; 3 School of Transportation and Logistics Engineering, Wuhan University of Technology, Wuhan 430063, China; yu.chen@whut.edu.cn; 4 School of Civil and Transportation Engineering, Beijing University of Civil Engineering and Architecture, Beijing 102616, China; menglingup@hotmail.com

Asphalt pavements are widely used in road networks worldwide, and their performance directly affects service quality, durability, and the maintenance needs of transportation systems. However, with the rapid growth of traffic volumes, increased axle loads, and the intensification of extreme climate events, the durability and sustainability of asphalt pavements have become pressing concerns for engineers and researchers alike. Conventional bitumen-based materials often struggle to meet the multifaceted performance requirements of modern highways, which include enhanced rutting resistance, fatigue endurance, aging resistance, and resilience under freeze–thaw and wet–dry cycles. To address these challenges, pavement engineering has increasingly turned to advanced material characterization, innovative modifiers, and novel modeling approaches to unlock improved long-term performance.

In recent decades, the research frontier has shifted from empirical mixture design toward performance-related investigations of the fundamental material properties that govern pavement behavior. This transition has been driven by the need for more mechanistic-empirical design methods, a greater emphasis on life-cycle sustainability, and the integration of advanced experimental tools with numerical simulations. Cutting-edge techniques such as discrete element modeling, viscoelastic and creep constitutive modeling, CT-based mesostructural analysis, and image processing-based adhesion evaluation are combined with laboratory testing to deepen our understanding of asphalt mixture components. Meanwhile, new materials such as polymers, fibers, nano-additives, and bio-based or resin alternatives are being explored to enhance rheological properties, extend service life, and mitigate environmental impacts. The result is a growing body of interdisciplinary knowledge that spans materials science, structural mechanics, and road engineering.

Despite these advances, knowledge gaps remain. Questions persist about the behavior of high-reclaimed asphalt pavement (RAP) mixtures at low temperatures, the mechanisms of fiber-binder interaction, the storage stability of polymer-modified binders, and the prediction of aging and emission characteristics under real-world conditions. Addressing these gaps requires bridging the scales from nano- and microstructural phenomena in binders and mastics, through meso-level aggregate gradation, to macro-level pavement response. Moreover, predictive models linking material chemistry to engineering performance remain under active development. This multi-scale and multi-perspective approach is essential for translating laboratory findings into field-ready applications.

Against this backdrop, the Special Issue “Performance-Related Material Properties of Asphalt Mixture Components (Second Edition)” brings together 14 peer-reviewed papers that collectively reflect the recent advances in this evolving field. The contributions represent a rich diversity of topics, ranging from recycled asphalt mixtures, advanced polymer and fiber modifiers, multi-scale testing of hot in-place recycling, performance enhancement of warm-mix asphalt with additives, predictive modeling of polymer-modified binders, nano-materials for rheological and aging improvements, comprehensive reviews on binder aging, rejuvenation strategies for aged binders, and new digital approaches for sustainability assessment in pavement construction. Together, these works provide valuable insights into how the intrinsic material properties of asphalt binders, mastics, mixtures, and subgrades directly affect pavement performance. They also highlight innovative methodologies that can serve as practical tools for research and engineering practice. By addressing both fundamental mechanisms and application-oriented solutions, the collection offers a balanced view of theoretical development and engineering practice. Importantly, it demonstrates the relevance of multi-scale approaches from the nano-scale interactions of polymer-modified asphalt binders to the meso-scale mechanical behavior of aggregates and the macro-scale performance of recycled mixtures. This holistic perspective reflects the ongoing paradigm shift in pavement engineering, where material design and structural performance are increasingly intertwined. Recycling has become one of the most impactful approaches for advancing sustainability in pavement engineering, reducing reliance on virgin materials while lowering costs and environmental burdens. The Special Issue highlights this area with an emphasis on high-dose reclaimed asphalt pavement (RAP) mixtures. Jiang et al. [1] investigated the performance of RAP mixtures in hot in-place recycling (HIR) using a balanced design framework. Their findings reveal the intricate trade-offs between rutting and cracking resistance as binder content increases, providing clear guidelines for optimizing mix design. This study underscores the importance of balanced performance criteria to ensure that recycled mixtures can be implemented at scale without compromising durability. Complementary insights are provided by Zhang et al. [2], who evaluated low-temperature cracking resistance in multi-scale HIR mixtures. By testing across binder, mastic, fine aggregate matrix (FAM), and mixture levels, they established strong correlations between fracture energy density at the meso-scale and overall mixture behavior. The results demonstrated that mixtures with over 90% RAP exhibited significant reductions in crack resistance, highlighting the need for careful RAP content control. In a related work, Zhang et al. [3] examined the macro- and micro-performance degradation of HIR mixtures with high RAP contents, combining in situ dynamic tensile testing with microstructural analyses. Their degradation model provided a predictive framework linking RAP content with crack resistance decline. Beyond conventional RAP recycling, Obaid et al. [4] explored warm-mix asphalt (WMA) containing RAP, reinforced with ceramic fibers. Their statistical modeling demonstrated significant improvements in rutting resistance, fatigue life, and moisture susceptibility when fibers were incorporated, particularly at intermediate temperatures. Khan et al. [5] extended this work by evaluating polyolefin/aramid fibers and pure aramid fibers in reinforced asphalt mixtures, showing how fiber reinforcement can shift mixture behavior from rutting resistance toward crack resistance depending on fiber type. These results collectively highlight how recycling, when combined with fiber or additive modification, can produce sustainable and high-performing mixtures.

Another major theme of the Special Issue is the development of advanced binders through polymer modification and predictive modeling. Zhang et al. [6] investigated the effect of linear and crosslinked POE-g-GMA copolymers on asphalt properties, showing that grafting significantly improves rutting resistance, compatibility, and storage stability, while enhancing resistance to oxidative aging. Wu et al. [7] advanced this direction by developing a BAS-BP neural network model for predicting the rheological performance of polymer-modified binders from chemical composition. Their results demonstrated that functional groups such as styrene and butadiene act as “phenotypic genes” correlating directly with rheological properties, enabling high-accuracy performance prediction. The role of base asphalt properties in determining storage stability was highlighted by Liu et al. [8], who investigated SBS-modified asphalt systems. Their study revealed how differences in the molecular weight distribution and microstructure of base asphalt strongly influence polymer segregation and phase separation during thermal storage. Such findings provide essential guidance for selecting compatible base binders to ensure long-term stability. Meanwhile, innovative fiber-based modifiers continue to expand the performance potential of polymer-modified asphalts. Li et al. [9] introduced nano-modified polyacrylonitrile (PAN) fibers produced via layer-by-layer self-assembly. When incorporated into SBS-modified asphalt, these fibers significantly enhanced viscoelasticity and rutting resistance due to stronger interfacial bonding. Hesami et al. [10] further explored nano-expanded perlite (NEP) as a modifier, demonstrating its ability to improve high-temperature performance and rutting resistance while maintaining low-temperature cracking resistance. Both studies confirm that nano-materials provide a promising pathway for performance enhancement, bridging microstructural modification with macroscopic mechanical benefits. In addition to material-level improvements, Zhang et al. [11] developed a low-temperature Performance Grade (PG) evaluation method using an 8 mm Dynamic Shear Rheometer test and a 2S2P1D-based time-temperature conversion to back-calculate Bending Beam Rheometer (BBR) indices. This approach circumvents the high cost and complexity of BBR testing and offers a more practical pathway for PG implementation, highlighting that innovation in test methods is as essential as innovation in materials.Aging remains one of the central challenges in asphalt pavement performance, directly affecting durability and life-cycle cost. Ahmad et al. [12] critically reviewed asphalt binder aging through multi-aspect analyses, synthesizing chemical, morphological, and rheological perspectives. Their work emphasized the need for holistic approaches to aging characterization, pointing out limitations in current methods and urging integrated, multi-scale analytical frameworks that can connect chemical, structural, and mechanical changes. Luo et al. [13] contributed experimental evidence by developing a zinc oxide/expanded vermiculite composite to improve the aging resistance of SBS-modified asphalt. Their results confirmed that the composite mitigates matrix and polymer degradation during thermal and photo-oxidative aging, achieving optimal resistance at 4% dosage. On the rejuvenation side, Xu et al. [14] proposed a two-component synchronous rejuvenator system combining triallyl isocyanurate and aromatic oil for aged SBS binders. By calibrating FTIR peak area ratios, they identified optimal dosages capable of restoring over 90% of the original rheological and microstructural performance of aged binders. This contribution demonstrates how targeted chemical strategies can enable the recycling of aged polymer-modified asphalts without compromising performance. Durability is also shaped by broader environmental and operational factors, including carbon emissions and digital optimization. Wu et al. [15] developed a novel deep learning–based approach to predict carbon emissions from pavers during asphalt construction, integrating SSDNN with the MOVES-NONROAD model. Their method achieved higher accuracy than conventional algorithms and supports low-carbon construction practices. Zhao et al. [16] expanded the digitalization frontier by proposing an automated gradation design model for stabilized waste gravel soil using a deep learning–PFC hybrid framework. Their approach demonstrated strong predictive accuracy and provides a template for integrating artificial intelligence into future pavement design.

The Special Issue “Performance-Related Material Properties of Asphalt Mixture Components (Second Edition)” brings together contributions highlighting the ongoing evolution of asphalt materials research. From recycling strategies and balanced design frameworks to innovative modifiers, aging mitigation, and digital modeling, the works showcase both breadth and depth of innovation. Notably, integrating experimental, chemical, and computational methods demonstrates the field’s growing interdisciplinarity, while the emphasis on sustainability reflects the pressing needs of modern infrastructure. Looking ahead, three directions emerge as particularly significant. First, advancing recycling technologies through balanced performance design and multi-scale analysis will be key to integrating high RAP contents without sacrificing durability. Second, modifiers at the molecular, fiber, and nano scales will continue to expand the performance envelope of asphalt binders and mixtures, especially in aging resistance and multi-temperature performance. Third, the digitalization of material design and environmental impact assessment will enable predictive, data-driven decision-making supporting engineering performance and sustainability.

We would like to sincerely thank all the authors for their high-quality contributions, the reviewers for their constructive feedback, and the editorial team of Materials for their invaluable support. This collective effort has made it possible to present a comprehensive and timely resource for the global pavement engineering community.
